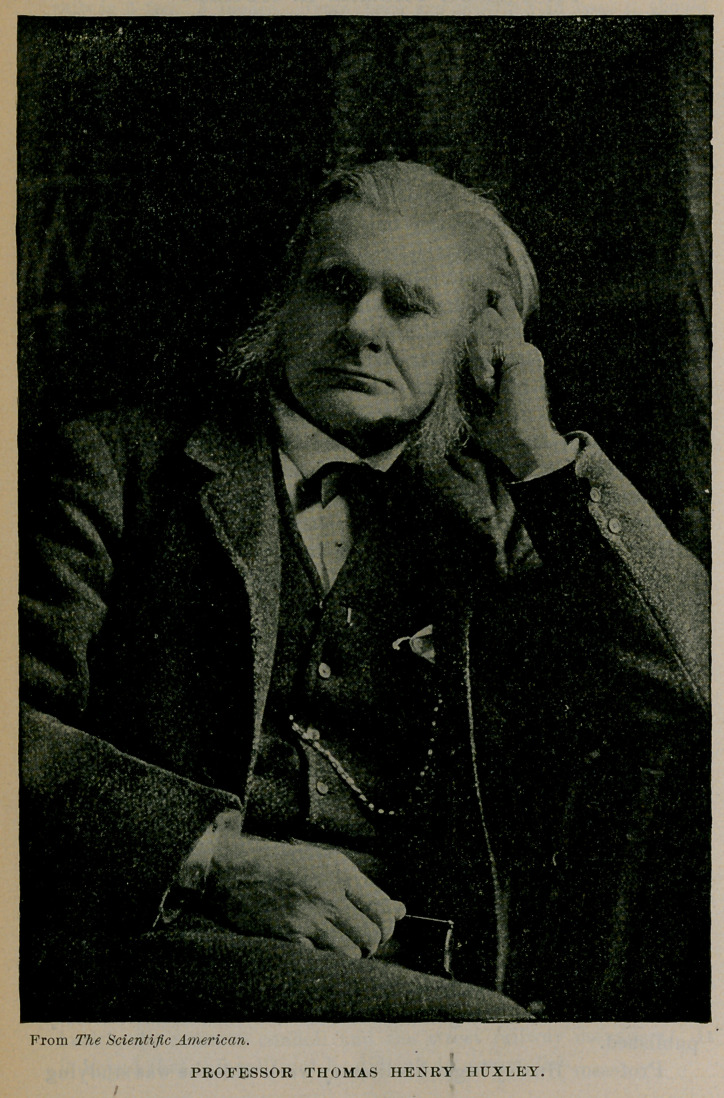# Professor Thomas H. Huxley

**Published:** 1895-08

**Authors:** 


### PROFESSOR THOMAS H. HUXLEY.

THE death of the eminent naturalist, Thomas Henry Huxley, that occurred at Eastbourne, England, June 29, 1895, deprives the world of the last but one of a group of the most illustrious scientists of his period. This group, composed of Darwin, Tyndall, Huxley and Spencer, have revolutionised thought and created a philosophy. They have established the doctrine of evolution in spite of an opposition that held sway for centuries and was so deeply rooted that only such a quartet of giant minds would have dared undertake to overthrow it. Darwin, Tyndall and Huxley are gone and only Herbert Spencer remains to round out the work of this phalanx of philosophy.

Professor Huxley was born at Ealing, Middlesex, Eng., May 4, 1825 ; hence, was just past seventy when he died. He was educated at Ealing school, where his father was a teacher, and at the age of seventeen he entered the Charing Cross medical school, London. Three years later he received the degree of bachelor of medicine, graduating with high honors in physiology. Soon afterward he entered the naval service as a medical officer and went with Stanley's expedition to the Eastern Archipelago. During the voyage he studied the natural history of the sea and devoted much attention to the Medusæ. After his return to England he was appointed professor of paleontology in the Government school of mines at London, and was also made Fullerian professor of physiology to the Royal Institution and examiner in physiology and comparative anatomy at the University of London. He went with Tyndall, in 1856, on his first trip to the Alps, and in 1858 he was made Croonian lecturer to the Royal society. About this time he gave numerous lectures on the relation of man to the lower animals. In 1863, he became professor of comparative anatomy at the Royal college of surgeons, where he remained seven years. In 1872, he was made lord rector of Aberdeen university, and in 1873 he was was chosen secretary of the Royal society. In 1881, he was made inspector-general of fisheries, succeeding Frank Buckland, and on the death of Mr. Spottiswoode, in 1884, Professor Huxley was elevated to the presidency of the Royal society. During a visit to America, in 1876, he delivered a series of lectures that attracted great attention and which were widely published.

Professor Huxley began his literary work while he was studying

From *The Scientific American.*

PROFESSOR THOMAS HENRY HUXLEY.

medicine at the Charing Cross hospital. His writings did much to popularise science. They include Oceanic Hydrozoa and Man's Place in Nature, 1863 ; Lessons on Comparative Anatomy, 1864 ; Lessons in Elementary Physiology, 1866 ; An Introduction to the Classification of Animals, 1869 ; Lay Sermons, Addresses and Reviews, 1870 ; Manual of the Anatomy of Vertebrated Animals, 1871 ; and Critiques and Addresses, 1873. Other works were on Origin of Species, More Criticism on Darwin and Administrative Nihilism, American Addresses, Physiography, The Crayfish, Science and Culture, and the Advance of Science in the Last Half Century.

The degrees conferred upon Professor Huxley were, M. D., Ph. D., LL. D., and D. C. L., but he was a member of the principal scientific societies of the world, from which he received many honors and decorations.

During the last ten years of his life he remained in practical retirement, so that of late the world has known less of him than formerly. For the beautiful picture we present of this great man we are indebted to the courtesy of the *Scientific American*, and we have gleaned many data of this sketch from the issue of that paper for July 13, 1895. Huxley had a contempt for autobiographies, but once sketched his own character, says the *Scientific American*, in the following words :

'That man has a liberal education who has been so trained in youth that his body is the ready servant of his will and does with ease and pleasure all the work that, as a mechanism, it is capable of ; whose intellect is a clear, cold logic engine, with all its parts of equal strength and in smooth working order, ready, like a steam engine, to be turned to any kind of work and spin the gossamers as well as forge the anchors of the mind ; whose mind is stored with a knowledge of the great and fundamental truths of nature and of the laws of her operations ; one who, no stunted ascetic, is full of life and fire, but whose passions are trained to come to halt by a vigorous will, the servant of a tender conscience ; who has learned to love all beauty, whether of nature or of art, to hate all vileness, and to respect others as himself. Such a one, and no other, has had a liberal education.'

The *Scientific American*, in concluding, most truthfully and delicately asseverates that "the world can add no higher tribute to the author of these words than to say that such a man was Thomas Henry Huxley."

## Figures and Tables

**Figure f1:**